# Study of the impact of *perilipin *polymorphisms in a French population

**DOI:** 10.1186/1477-5751-5-10

**Published:** 2006-07-12

**Authors:** Aline Meirhaeghe, Séverine Thomas, Frédéric Ancot, Dominique Cottel, Dominique Arveiler, Jean Ferrières, Philippe Amouyel

**Affiliations:** 1 INSERM, U744, Lille; Institut Pasteur de Lille, Lille; Université de Lille 2, Lille, France; 2 Department of Epidemiology and Public Health, Faculty of Medicine, Strasbourg, France; 3 INSERM, U558, Faculté de Médecine, Toulouse, France

## Abstract

**Background:**

Perilipins are proteins localized at the surface of the lipid droplet in adipocytes, steroid-producing cells and ruptured atherosclerotic plaques playing a role in the regulation of triglyceride deposition and mobilization. We investigated whether perilipin gene polymorphisms were associated with obesity, type 2 diabetes, and their related variables (anthropometric variables, plasma leptin, lipids, glucose and insulin concentrations) in a cross-sectional random sample of 1120 French men and women aged 35 to 65 years old, including 227 obese (BMI ≥ 30 kg/m^2^) and 275 type 2 diabetes subjects.

**Results:**

Among 7 *perilipin *polymorphisms tested, only 2 (rs4578621 and rs894160) of them were frequent enough to be fully investigated and we genotyped the sample using the PCR-RFLP method. No significant associations could be found between any of these polymorphisms and the studied phenotypes.

**Conclusion:**

The rs4578621 and rs894160 polymorphisms of the perilipin gene are not major genetic determinants of obesity and type 2 diabetes-related phenotypes in a random sample of French men and women.

## Background

Perilipins are phosphorylated proteins in adipocytes localized at the surface of the lipid droplet in adipocytes, steroid-producing cells and ruptured atherosclerotic plaques [1-4]. These proteins are essential in the regulation of triglyceride deposition and mobilization [5-7]. When protein kinase A is activated, perilipin A becomes phosphorylated and translocates away from the lipid droplet, which allows hormone-sensitive lipase to hydrolyse the adipocyte triglyceride core [8,9]. Therefore, perilipin A increases cellular triglyceride storage by decreasing the rate of triglyceride hydrolysis. Mice knockout for the perilipin gene are lean, have increased basal lipolysis and are resistant to diet-induced obesity [10,11]. However, these mice also develop glucose intolerance and insulin resistance more readily, probably due to the elevated levels of non-esterified fatty acids.

Several studies determined the level of perilipin expression according to obesity status. Two studies found obese subjects displayed lower levels of perilipin than lean individuals [7,12] whereas another study showed perilipin mRNA and protein were elevated in obese subjects [13]. Qi *et al*. previously showed that polymorphisms in the perilipin locus were associated with obesity-related phenotypes in American and Spanish White women [14,15]. Moreover, they showed that a particular haplotype was associated with increased obesity risk in Malays and Indians, but not in Chinese [16]. In this study, we examined the genetic variability of the perilipin gene and possible associations with obesity, type 2 diabetes and related phenotypes in a French random sample of population.

## Results

Table 1 describes the genotyping conditions and frequencies of the 7 tested *PLIN *SNPs (issued from the NCBI dbSNP database). DNA from 90 individuals issued from the population study was used to estimate the SNP frequencies. The rs8179072 (A386V), rs8179070 (R274W), rs3743373 (E293K) SNPs were not detected at all. Only 1 heterozygote for both the rs6496589 (Pro194Ala) and rs8179071 (Ser348Leu) SNPs could be found (rare allele frequency = 0.6 %). Therefore we did not pursue the genotyping of these SNPs. The rare allele frequencies of the rs4578621 (5'UTR -1234 C>G) and rs894160 (intron 6) SNPs were 7.6 and 30 % respectively. Therefore, we genotyped these two SNPs in the whole population study (n = 1120). There were 958 (85.5%) GG, 153 (13.7 %) GA, 9 (0.8%) AA and 556 (49.6%) GG, 470 (42.0%) GA and 94 (8.4%) AA for the rs4578621 and rs894160 SNPs respectively. These frequencies were not different from the expected frequencies under Hardy-Weinberg equilibrium.

**Table 1:** Description of *PLIN *SNPs, primers, PCR conditions and restriction enzymes.

SNP	Primers	PCR (bp)	MgCl_2 _(mM)	Ann. temp.	RE	Size (bp) for the wt allele	Size (bp) for the mut allele	MAF (%)
rs4578621	F : CAAGCTGGGTGCACTGGC R : GAGAAATAGAGGAATTAACC	185	0.9	58	*Mnl*I	33+152	33+62+90	7.6
rs6496589	F : CTGCCAACACTCG AGCTG R : ACCTGACTCTTCCTTGTCT	110	1	60	*Pst*I	72+28	no cut	0.6
rs894160	F1 : GCTGAGACTGAGTCACATGC R1 : GCTGAGACTGAGTCACATGC	403	0.5	60	-	-	-	-
	F2 : CTGTTTGTGGGGCTCCCTCG R2 : CCTCCCAGATCTTTTAAGAG	126 (nested)	0.5	52	*Xho*I	126	20+106	30.0
rs8179070	F : CAGCTATCTGCTGCCATC R : CTCACTGAACTTGTTCTCC	219	0.5	58	*Msp*I	75+144	219	ND
rs3743373	F : CAGCTATCTGCTGCCATC R : CTCACTGAACTTGTTCTCC	219	0.5	58	*BseR*I	96+49+45+21+5+3	96+94+21+ 5+3	ND
rs8179071	F : GTAGCAGCCCTGCCAGGCC R : CCGGCACGTAATGCACCAC	248	0.5	69	*BstX*I	66+182	66+129+53	0.6
rs8179072	F : GTAGCAGCCCTGCCAGGCC R : CCGGCACGTAATGCACCAC	248	0.5	69	*Hpy188*III	248	80+168	ND

F : forward, R : reverse, wt allele : wild-type (common) allele, mut allele : mutant (rare) allele, Ann. : annealing, temp. : temperature, RE: restriction enzyme, MAF: minor allele frequency.

The SNP allele frequencies were determined in 90 individuals.

We compared the genotype distribution of the two SNPs according to BMI categories (normal weight: BMI<25 kg/m^2^, overweight : 25≤BMI<30 kg/m^2 ^and obese : BMI≥30 kg/m^2^) and diabetes status in men and women separately (table 2). No significant differences in the SNPs frequency could be detected between BMI or diabetes categories.

**Table 2:** Genotype distribution of the *PLIN *rs4578621 and rs894160 SNP according to BMI categories and diabetes status

	**rs4578621**			**rs894160**			
		
	**CC**	**CG+GG**	***p***	**GG**	**GA**	**AA**	***p***
		
	**Men**			**Men**			
		
BMI<25 kg/m^2 ^(228)	194 (85.1)	34 (14.9)		109 (47.8)	97 (42.5)	22 (9.7)	
25≤BMI<30 kg/m^2 ^(238)	204 (85.7)	34 (14.3)	ns	113 (47.5)	103 (43.3)	22 (9.2)	ns
BMI≥30 kg/m^2 ^(104)	91 (87.5)	13 (12.5)		58 (55.8)	36 (34.6)	10 (9.6)	
	
Non-diabetic (397)	337 (84.9)	60 (15.1)		186 (46.9)	168 (42.3)	43 (10.8)	
Diabetic (151 M/160 W)	127 (84.1)	24 (15.9)	ns	82 (51.3)	62 (38.7)	16 (10.0)	ns
	
	**Women**			**Women**			
		
BMI<25 kg/m^2 ^(249)	216 (86.8)	33 (13.2)		129 (51.8)	97 (39.0)	23 (9.2)	
25≤BMI<30 kg/m^2 ^(172)	145 (84.3)	27 (15.7)	ns	87 (50.6)	79(45.9)	6 (3.5)	ns
BMI≥30 kg/m^2 ^(123)	103 (83.7)	20 (16.3)		58 (47.2)	54 (43.9)	11 (8.9)	
	
Non-diabetic (439)	382 (87.0)	57 (13.0)		220 (50.1)	188 (42.8)	31 (7.1)	
Diabetic (108 M/115 W)	91 (84.3)	17 (15.7)	ns	66 (57.4)	39 (33.9)	10 (8.7)	ns

Data are n (%). Among the 1120 individuals genotyped, data on BMI or diabetes status were missing for few subjects. M: men, W: women.

Table 3 and 4 show the impact of the PLIN rs4578621 and rs894160 SNPs respectively on anthropometric and biological variables in men and women separately. The means of anthropometric variables (weight, BMI, waist and hip circumferences, or waist-to-hip ratio), plasma lipid (plasma cholesterol, HDL-cholesterol, LDL-cholesterol, triglycerides) or insulin concentrations were not statistically different between genotype groups neither in men nor in women. Men carrying the rare *AA *genotype of the rs894160 SNP had lower fasting plasma glucose levels than *GG *subjects (5.27 ± 0.64 vs. 5.56 ± 0.90 mmol/L or 1.65 ± 0.12 vs. 1.71 ± 0.15 log-transformed values for *AA *vs *GG *men respectively, p = 0.04 when adjusted for covariates).

**Table 3:** Impact of the *PLIN *rs4578621 SNP on clinical variables in men and women separately

	**Men**		**Women**	
		
	**CC** 487	**CG+GG** 81	**CC** 462	**CG+GG** 80
	
Weight, kg	79.9 ± 13.6	79.6 ± 12.9	68.1 ± 14.5	70.8 ± 15.2
BMI, kg/m^2^	26.7 ± 4.2	26.0 ± 3.6	26.4 ± 5.4	27.3 ± 5.9
Waist, cm	96.2 ± 11.0	95.2 ± 9.8	85.3 ± 13.8	87.9 ± 15.0
Hip circ., cm	101.5 ± 7.4	101.6 ± 6.8	103.5 ± 11.6	105.0 ± 12.7
WHR	0.95 ± 0.07	0.94 ± 0.06	0.82 ± 0.08	0.83 ± 0.08
Leptin, ng/mL^†^	9.48 ± 7.75	18.16 ± 6.59	24.08 ± 13.90	24.17 ± 14.98
	
Cholesterol, mmol/L	5.88 ± 1.06	5.92 ± 0.94	5.92 ± 1.11	6.08 ± 1.13
HDL-chol., mmol/L	1.34 ± 0.42	1.31 ± 0.36	1.67 ± 0.49	1.66 ± 0.49
LDL-chol., mmol/L	3.86 ± 1.02	3.89 ± 0.86	3.71 ± 1.04	3.90 ± 1.08
Triglycerides, mmol/L ^†^	1.64 ± 1.38	1.69 ± 1.52	1.19 ± 0.80	1.25 ± 1.37
Glucose, mmol/L ^†^	5.66 ± 1.37	5.52 ± 1.45	5.33 ± 1.28	5.56 ± 1.41
Insulin, μU/mL ^†^	12.37 ± 9.35	11.95 ± 10.01	11.63 ± 6.00	12.06 ± 5.84

^† ^These variables were log-transformed to obtain normal distributions. Circ. : circumference. WHR : waist-to-hip ratio. Chol. : cholesterol. Among the 1120 individuals genotyped, data on several variables were missing for few subjects.

**Table 4:** Impact of the *PLIN *rs894160 SNP on clinical variables in men and women separately

	**Men**			**Women**		
		
	**GG** 281	**GA** 235	**AA** 54	**GG** 272	**GA** 231	**AA** 39
		
Weight, kg	80.2 ± 14.1	79.2 ± 12.8	81.1 ± 13.2	67.5 ± 14.1	69.9 ± 14.6	67.9 ± 16.8
BMI, kg/m^2^	26.9 ± 4.3	26.3 ± 4.0	26.3 ± 3.7	26.2 ± 5.3	27.0 ± 5.4	26.7 ± 7.1
Waist, cm	96.5 ± 11.4	95.5 ± 10.2	96.3 ± 10.3	84.7 ± 13.4	87.0 ± 14.1	84.3 ± 16.9
Hip circ., cm	101.7 ± 7.5	101.4 ± 7.3	101.4 ± 7.1	102.9 ± 11.1	104.6 ± 11.8	103.7 ± 15.5
WHR	0.95 ± 0.07	0.94 ± 0.07	0.95 ± 0.07	0.82 ± 0.08	0.83 ± 0.08	0.81 ± 0.07
Leptin, ng/mL^†^	9.60 ± 7.90	9.18 ± 7.55	8.24 ± 6.19	23.12 ± 13.72	25.26 ± 14.52	23.79 ± 13.28
	
Cholesterol, mmol/L	5.93 ± 1.10	5.83 ± 1.00	5.86 ± 0.90	5.90 ± 1.18	6.01 ± 1.05	5.93 ± 1.11
HDL-chol., mmol/L	1.33 ± 0.41	1.37 ± 0.41	1.24 ± 0.37	1.65 ± 0.51	1.68 ± 0.47	1.69 ± 0.43
LDL-chol., mmol/L	3.90 ± 1.05	3.79 ± 0.96	3.99 ± 0.85	3.69 ± 1.10	3.80 ± 0.99	3.68 ± 1.05
Triglycerides, mmol/L^†^	1.71 ± 1.42	1.57 ± 1.31	1.68 ± 1.66	1.21 ± 0.87	1.19 ± 0.97	1.16 ± 0.77
Glucose, mmol/L^†^	5.56 ± 0.90	5.49 ± 0.85	5.27 ± 0.64*	5.40 ± 1.41	5.42 ± 1.71	5.64 ± 1.55
Insulin, μU/mL^†^	12.52 ± 9.48	11.93 ± 8.16	12.83 ± 13.62	11.47 ± 5.86	11.83 ± 5.87	12.43 ± 7.32

*crude p < 0.03 or p < 0.04 when adjusted for age, BMI, smoking and alcohol consumptions with a recessive model (adjusted means 5.51 ± 0.05 for GG vs 5.52 ± 0.05 for GA vs 5.28 ± 0.11 for AA subjects). ^† ^These variables were log-transformed to obtain normal distributions. Circ. : circumference. WHR : waist-to-hip ratio. Chol. : cholesterol. Among the 1120 individuals genotyped, data on several variables were missing for few subjects.

Between the two SNPs, the linkage disequilibrium D' was 0.87 (p < 0.0001), the G allele of the rs4578621 SNPS being associated with the A allele of the rs894160 SNP, and the r^2 ^was 0.15. Therefore, three haplotypes covered 99 % of the possible haplotypes (haplotype frequencies, CG: 0.70, CA: 0.22, GA: 0.07). No significant association could be found between haplotypes and the phenotypes studied (data not shown).

## Discussion

In the present study, we assessed the impact of the genetic variability of the PLIN gene on obesity, type 2 diabetes and related phenotypes in a sample of men and women issued from a French random sample of population (n = 1120). The genotyping of two common SNPs (rs4578621 and rs894160, frequency = 7.6 and 30 % respectively) were performed. No significant associations could be detected between these SNPs and the anthropometric variables, plasma leptin, lipids, glucose and insulin concentrations, even when using haplotype analyses.

The only significant association we found in our study was that men carrying the rare *AA *genotype of the rs894160 SNP (also called11482 G>A) had significant lower fasting plasma glucose levels than *GG *subjects but this difference was statistically borderline (p = 0.04) and clinically marginal (around 5% lower) and therefore this association is not clinically of great consequence and probably obtained by hazard. Moreover, this polymorphism was not associated with type 2 diabetes neither in men, nor in women in our study. Although the 11482 G>A SNP seems to have a functional impact on the perilipin protein activity as Mottagui-Tabar *et al*. reported that the A allele was associated with enhanced basal and noradrenaline-induced lipolysis in human subcutaneous fat cells [7], it does not seem to have a major impact in human French populations.

Other studies have previously described associations between *PLIN *SNPs and obesity-related phenotypes, especially the rs894160 (11482 G>A) SNP. Qi L *et al*. showed that a *PLIN *specific haplotype was associated with an increased risk of obesity in Malays, Indians and Whites (odds ratio around 1.7) [14-16]. In Whites, the *PLIN *11482 G>A was associated with obesity risk in women but not in men. We were not able to reproduce this association in our French study. Although our study bears on a large number of subjects (573 men, 547 women), its statistical power may still be insufficient to detect small associations. This hypothesis is supported by an *a posteriori *calculation which indicates that according to the observed allele distribution, the sample size has sufficient statistical power (1-β≥ 80%) to detect an odds ratio above 2.4 and 2.1 for obesity and 2.1 and 1.8 for type 2 diabetes for the rs4578621 and rs894160 SNPs respectively. Therefore, only major associations could be detected. Moreover, we can not exclude that other *PLIN *SNPs that we did not explore, taken individually or in haplotype combinations, might be associated with obesity phenotypes.

## Conclusion

In conclusion, the *PLIN *rs4578621 and rs894160 polymorphisms do not seem to be major genetic determinants of obesity and type 2 diabetes risk in French men and women. Other larger studies and/or other *PLIN *SNPs may need to be studied to conclude definitely about the impact of the PLIN gene variability on metabolic diseases.

## Methods

### Study subjects

Participants were recruited within the framework of the WHO-MONICA population survey conducted from 1995 to 1997 in the Urban Community of Lille in the North of France. The sample included subjects aged 35–65 years, randomly selected from the electoral rolls to obtain 200 participants for each gender and 10-year age group [17,18]. A total number of 601 men and 594 women was recruited. To our knowledge, no individuals were related. The Ethical Committee of Lille University Hospital (CHRU de Lille) approved the protocol.

After signing an informed consent, participants were administered a standard questionnaire and physical measurements were made by a trained nurse. The level of physical activity was defined as: walking or riding 15 min or more per day, and/or lifting or carrying heavy objects at work daily, and/or doing sport or physical exercise more than 2 hours a week. Current cigarette smokers were defined as subjects reporting at least one cigarette per day. Total alcohol intake was expressed as the sum of ml alcohol per week from wine, beer, cider and spirits.

The anthropometric measurements included body weight, waist and hip circumferences. BMI was calculated according to the Quetelet equation. Blood pressure was measured on the right arm, with the subject in a sitting position and after a minimum 5-min rest, using a standard mercury sphygmomanometer. The mean value of two consecutive blood pressure readings was taken into account. From this sample, 232 subjects were obese (BMI ≥ 30 kg/m^2^).

Individuals with type 2 diabetes (n = 275) were identified on the basis of a medical diagnosis and/or fasting glycaemia ≥ 7 mmol/L (1.26 g/L) and/or on the existence of a specific treatment or diet [19] in the Lille sample and in two other representative French samples (Strasbourg, Toulouse) participating to the risk factor surveys of the WHO-MONICA project. Control subjects for type 2 diabetes had fasting glycaemia < 6.1 mmol/L (1.10 g/L) and had no specific treatment or diet for type 2 diabetes.

### Laboratory methods

A blood sample of 20 mL was drawn on disodium EDTA after the subjects had fasted for at least 10 hours. Lipid and lipoprotein levels were measured in a central laboratory Purpan Hospital Biochemical Laboratory (Toulouse). The quality of biological measures was assessed within the framework of the MONICA Project. Glucose was measured by a standard glucose hexokinase method (DuPont Dimension, Brussels, Belgium). Plasma insulin was measured by radio-immunoassay (Medgenix Diagnostics, Brussels, Belgium). Plasma total cholesterol and triglyceride levels were measured by enzymatic methods (DuPont Dimension Brussels, Belgium). High density lipoprotein (HDL) cholesterol was measured after sodium phosphotungstate/magnesium chloride precipitation (Boehringer Mannheim, Mannheim, Germany). Low density lipoprotein (LDL) cholesterol was calculated with the Friedewald equation. Plasma leptin levels were measured by radio-immunoassay (Human leptin RIA kit, Wak-Chemie, Medical GmbH, Germany).

### DNA isolation and genotyping

Genomic DNA was extracted from white blood cells isolated from 20 ml of whole blood using a commercially available DNA isolation kit (DNA extraction kit, Stratagene, La Jolla, CA, USA). Genomic DNA was available for 1155 subjects. The PCR and genotyping conditions for the SNPs (single nucleotide polymorphisms) are described in table 1. A total of 1120 subjects were genotyped for the *PLIN *rs4578621 and rs894160 SNPs.

### Statistical analyses

Chi-square analysis or Fisher exact tests were used to compare genotype and allele distributions between groups. Comparison of differences among genotype groups were tested using a general linear model (proc GLM). Adjustment variables were : age, alcohol, smoking, and physical activity for the anthropometric variables and age, BMI, alcohol, and smoking for the biological variables. A dominant model was tested for the rs4578621 SNP due to the low number of homozygotes for the rare allele. A dominant and a recessive model were tested for the rs894160 SNP. Analyses were performed with the SAS statistical software release 8 (SAS Institute Inc, Cary, NC). Haplotype analyses were based on the maximum likelihood model described in and linked to the SEM algorithm [20,21] and performed using the software developed by the INSERM U525, Paris, France (available at ). Statistical significance was defined at the 5% level. Power calculation was done with the Epi Info 6.04 software available on . The statistical power (1-β) was set above 80%.

## Competing interests

The author(s) declare that they have no competing interests.

## Authors' contributions

FA, ST genotyped the population samples. AM analyzed the data and wrote the paper. DC, DA, JF and PA enrolled the participants and contributed to writing the paper.
